# Duodenal transection following a seat belt injury: A case report

**DOI:** 10.1016/j.ijscr.2022.107272

**Published:** 2022-06-01

**Authors:** Hassan Shah, Belal Nedal Sabbah, Badr Ahmed Elwy, Tarek Ziad Arabi, Ahmad Nedal Sabbah, Syed Yousaf Shah

**Affiliations:** a College of Medicine, Alfaisal University, Riyadh, Saudi Arabia; b Department of Pediatric Surgery, King Salman Hospital, Riyadh, Saudi Arabia

**Keywords:** Duodenal transection, Trauma surgery, Duodenal injury, Duodenal perforation

## Abstract

**Introduction and importance:**

The rare presentation of duodenal injuries has led to a lack of guidelines for managing and diagnosing such cases. In most duodenal injuries, intramural hematoma and perforation are seen; however, complete resection of the duodenum is rare, which is seen in our case.

**Case presentation:**

We report a rare case of a 6-year-old boy who suffered from a complete isolated duodenal transection at the pylorus and a 90% transection at D3 and D4 following a seat-belt injury. The surgeon performed a primary anastomosis for the first part of the duodenum with pyloric exclusion. Then, primary repair with controlled fistula for the second transection at D3 and D4 and a gastrojejunostomy were performed. After further management, the patient was discharged with no further complaints.

**Clinical discussion:**

Due to the retroperitoneal location of the duodenum, it is challenging to diagnose a duodenal injury. CT scan with contrast is considered the best diagnostic tool in the case of a duodenal injury. Treatment of duodenal injuries depends on the type of injury and the present level of damage. It is imperative to differentiate between a duodenal hematoma, a duodenal perforation, or a duodenal transection as the management for each complication differs.

**Conclusion:**

No official guidelines have been set in the case of management or diagnosis of duodenal transection. Based on our experience with this patient and similar literature, guidelines for managing and diagnosing duodenal transection should be set, and further studies on the matter are warranted.

## Highlights

• Duodenal injury may be caused by various etiologies, including automobile accidents.
• Duodenal injury should remain a differential diagnosis in pediatric trauma patients.
• CT scan with contrast is the best imaging modality for isolated duodenal injuries.
• Primary anastomosis remains the mainstay treatment for duodenal transection.

## 1 Introduction

Blunt abdominal trauma in children rarely results in duodenal injury [1]. Pediatric duodenal injury accounts for less than 5% of pediatric intrabdominal injuries [2]. The rare presentation of duodenal injuries has led to a lack of guidelines for managing and diagnosing such cases. Duodenal injuries typically manifest with abuse, road traffic accidents, falls, or handlebar injuries [3]. In most duodenal injuries, intramural hematoma and perforation are seen; however, complete resection of the duodenum is extremely rare [3], which is what is seen in our case. The most common presentation of duodenal injury is abdominal pain, sometimes accompanying symptoms of peritonitis should the patient present late [4].

Diagnostics in case of blunt abdominal trauma include X-rays, abdominal ultrasound, and laboratory tests [5]. X-rays may show the presence of free air in the abdomen, while abdominal ultrasound can assess the status of the duodenum and any associated injuries [5]. The diagnosis of a duodenal injury is challenging and often delayed due to the retroperitoneal anatomical location and infrequent presentations to clinicians. A delay in diagnosis is associated with a higher rate of complications [6]. In the case of a suspected duodenal injury, an enhanced abdominal computed tomography (CT) scan should be done to confirm or exclude the presence of air or fluid in the retroperitoneal space [7].

We report a case of a complete isolated duodenal transection at the pylorus, along with a 90% transection at the second and third part of the duodenum, following a seat-belt injury. This case report is reported in line with the SCARE criteria [8].

## 2 Case presentation

**Fig. 1:**
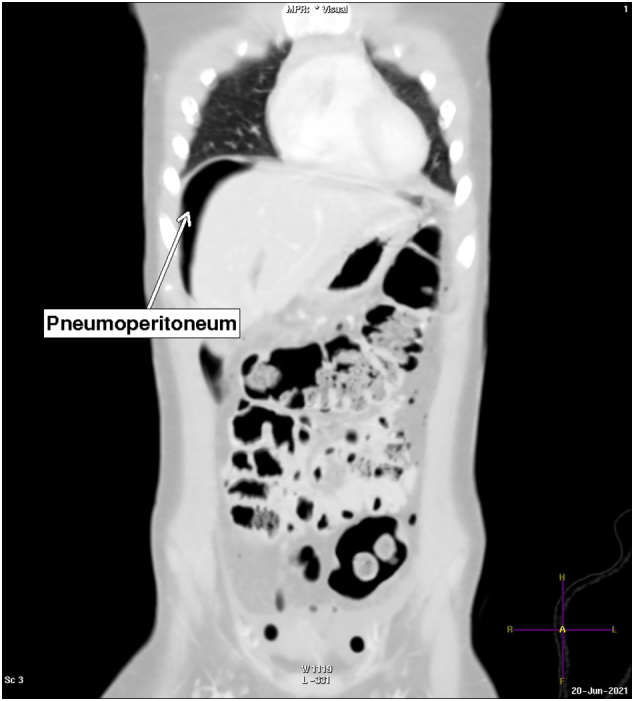
Coronal CT image in a lung window setting, free air around the liver margin and small specks of air foci on both sides of the abdomen.

**Fig. 2:**
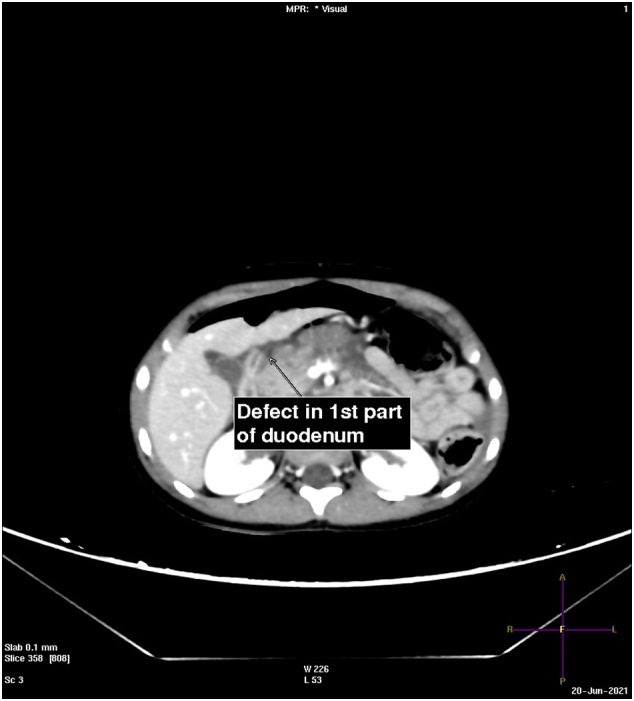
Axial CT with IV contrast, perforation noted in 1st part of the duodenum (arrow).

**Fig. 3:**
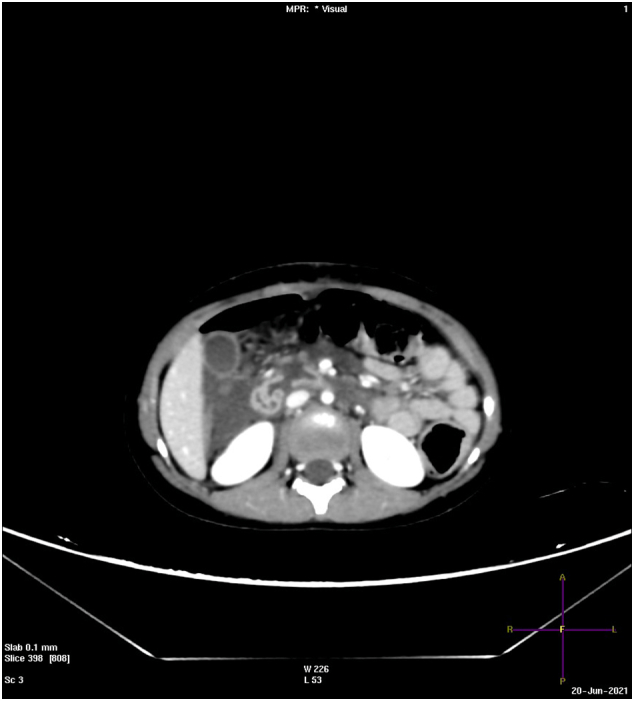
Axial CT with IV contrast; perforation seen in 3rd part of duodenum with free fluid.

A 6-year-old boy who was the front passenger in an automobile accident was brought to the hospital by an ambulance and presented with vomiting and abdominal pain. The patient underwent a pan CT without contrast, showing small right-sided parietal subdural hematoma and free fluid in the peritoneal cavity. The patient was then transferred to King Salman Hospital for further management. On arrival at the hospital, the patient was fully conscious, tachycardic, and had generalized abdominal pain. After initial resuscitation, an abdominal CT scan with contrast showed free fluid in the peritoneal cavity with pneumoperitoneum [Fig. 1]. All solid viscera were intact, but there was a radiological impression of duodenal perforation [Fig. 2, Fig. 3]. The patient did not have a history of drug use or a significant family history.

**Fig. 4:**
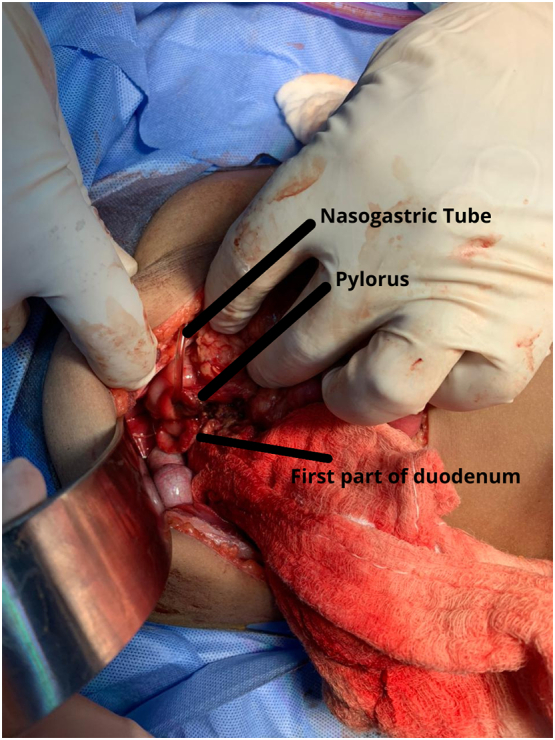
Transection of the pylorus and the 1st part of the duodenum can be seen. A nasogastric tube can be seen exiting the pylorus.

**Fig. 5:**
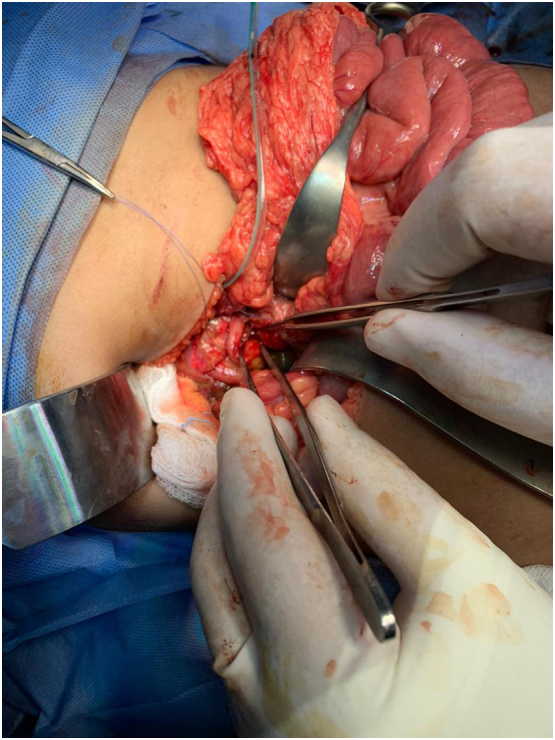
Perforation of the 3rd part of the duodenum proximal to the duodenojejunal junction.

The patient underwent exploratory surgery immediately by the attending surgeon. After initial optimization, the intra-operative findings consisted of approximately 500 ml of free intra-abdominal blood and a nasogastric tube (NGT) protruding outside the stomach [Fig. 4, Fig. 5]. It was found that the first duodenal segment was completely transected from the pylorus with healthy margins, and a 90% transection between the 3rd part of the duodenum (D3) and 4th part of the duodenum (D4) was seen with healthy margins. After abdominal irrigation with normal saline, primary anastomoses for the first part of the duodenum with pyloric exclusion were performed. Then, primary repair with controlled fistula for the second transection at D3 and D4 and a gastrojejunostomy were performed. Three abdominal drains were placed, one subhepatic, one paraduodenal, and one pelvic. A size 10 Foley's catheter was placed for a controlled fistula in the second part of the duodenal repair.

The patient was transferred postoperatively to the pediatric intensive care unit (PICU) at King Saud Medical City. On arrival, he had some confusion and vision problems, for which a CT scan of the brain was performed. The CT scan showed a stable small right-sided parietal subdural hematoma. The patient had also developed a partial collapse of the right upper lobe, which improved with assisted ventilation. The patient was treated by maintaining nothing by mouth (NPO), total parenteral nutrition (TPN), intravenous (IV) antibiotics, and replacing the drain fluid. The total drain output of all the four drainages was initially between 210 and 230 ml/day, which gradually decreased over ten days. Likewise, NGT output was around 250 ml/day and gradually reduced to a minimum over ten days. During the course of the illness, the patient had elevated serum amylase on the third postoperative day. In addition, an ultrasound (US) abdomen was performed, showing minimal peri-pancreatic fluid with pancreatic thickening and mild dilatation of the pancreatic duct, suggestive of acute pancreatitis. The patient improved clinically with conservative treatment within a week with a reduction in serum amylase to normal levels.

The contrast meal follow-through was performed with no evidence of contrast leakage or obstruction. Oral feeding resumed ten days postoperatively. The patient had a laparotomy wound infection with wound dehiscence, which was repaired after the infection resolved and the wound culture and sensitivity swab was negative. The patient was discharged with no further complaints.

## 3 Discussion

Isolated duodenal injuries are a rare but potentially dangerous type of pediatric trauma. Only 42 cases of duodenal injury were reported in a pediatric trauma center in the United States over ten years [9]. Due to the retroperitoneal location of the duodenum, it is challenging to diagnose a duodenal injury [7]. Patients with duodenal injury usually present with nausea, vomiting, and abdominal pain [7]. However, these findings are quite general and do not help differentiate the type of duodenal injury [7]. Physical exam findings will often be non-specific, and plain radiographs will not show free air caused by a duodenal perforation [9]. Duodenography is generally not a viable option, particularly in a trauma setting [9]. However, a CT scan with contrast is considered the best diagnostic tool in the case of a duodenal injury [9]. The presence of free air or extravasated contrast in a CT scan is an indication to operate on the patient [9].

Duodenal injuries can be caused by motor vehicle collisions, falls, handlebar impact, physical abuse, falling objects, and unknown insults [10]. Traumatic duodenal perforations are caused by either shearing forces or increased intraluminal pressure causing a “blowout” [10]. In contrast, a traumatic duodenal hematoma is caused by compression of the duodenum against the vertebral column [10]. Duodenal hematomas are considered the most common type of injury caused by blunt trauma in children [10].

Treatment of duodenal injuries depends on the type of injury and the present level of damage. It is imperative to differentiate between a duodenal hematoma and a duodenal perforation as the management for each complication differs [10]. Duodenal hematomas are usually managed non-operatively 89% of the time [10]. The non-operative treatment of duodenal hematomas includes nasogastric decompression and parenteral nutrition. However, this approach is associated with an increased hospital stay and gastric outlet obstruction [9]. Surgical exploration is indicated if there are either peritoneal or radiological signs [9].

On the other hand, duodenal perforations are treated with immediate surgical exploration. As a result, 80% of duodenal perforations are treated with primary repair, while 20% to 28% require more complex procedures [7]. Diversion and drainage are recommended for severe duodenal injuries or injuries with a delayed diagnosis [7]. Surgical treatment of complex cases consists of a primary suture of the defect, a pyloric exclusion, and a gastroenterostomy [7]. In our case, this procedure was also done, along with a controlled fistula to allow for duodenal drainage. Duodenal drainage is recommended in complex cases [7] because it allows for early detection and control of the duodenal fistula [11].

In the case of complete transection of the duodenum, primary suturing is still viable if there is minimal tissue loss, the ampulla of Vater is not involved, and the damage can be closed without tension [7]. If excessive tissue damage is present along with a completely transected duodenum, approximation of the duodenal ends is impossible [11]. In such cases, an antrectomy, duodenal stump closure, and a Billroth II gastrojejunostomy should be done [11]. However, in the case of an injury distal to the ampulla of Vater, a duodenojejunal Roux-en-Y anastomosis along with the closure of the duodenum is deemed appropriate [11]. Duodenopancreatectomy is indicated as the procedure of choice in the case of duodenal injury associated with either pancreatic bleeding, common hepatic duct injury, or pancreatic duct injury [7].

Since duodenal injuries are infrequent and difficult to diagnose, they are associated with a delay in treatment [9]. It is reported that more than 24 h of operative delay increases the complication rate from 29% to 43% and the mortality rate from 11% to 40% [9].

Moreover, corresponding intra-abdominal injuries determine morbidity and mortality [12]. The increased risk of mortality is the reason to exclude other associated injuries. For example, in patients with duodenal injury, 42% have associated pancreatic injuries, 29% have liver injuries, and 17% have splenic injuries [12]. Mortality in isolated duodenal injury is generally lower and is typically caused by uncontrolled sepsis, duodenal dehiscence, and multiple organ dysfunction syndrome [13].

## 4 Conclusion

Isolated duodenal injuries are challenging to diagnose because of their rare occurrence, location, and non-specific signs and symptoms. Early diagnosis is crucial as delay in diagnosis is associated with increased morbidity and mortality. The treatment depends on the type and severity of injury. Duodenal hematomas are generally treated conservatively. In contrast, the mainstay of treatment for duodenal perforation is a primary anastomosis, with additional procedures done as required. However, no official guidelines have been set in the case of management or diagnosis of duodenal transection. Based on our experience with this patient and similar literature, guidelines for managing and diagnosing duodenal transection should be set, and further studies on the matter are warranted.

## Availability of data and material

Not applicable.

## Code availability

Not applicable.

## Consent to participate

Not applicable.

## Provenance and peer review

Not commissioned, externally peer-reviewed.

## Sources of funding

This study did not receive funding from any source.

## Ethical approval

Patient anonymity is maintained throughout this manuscript, and consent was obtained for publication from the patient's parents.

## Consent for publication

Written informed consent was obtained from the patient's family for publication of this case report and accompanying images. A copy of the written consent is available for review by the Editor-in-Chief of this journal on request.

## Author contribution

HS, BNS, BAE, TZA, ANS, drafted the manuscript SYS contributed to reviewing and finalizing the manuscript. He also provided the imaging findings and their interpretation for the case presentation section. All authors reviewed the manuscript for intellectual content and approved the submission.

## Research registration

N/A.

## Guarantor

Syed Yousaf Shah, MBBS, MRCS.

## Declaration of competing interest

The authors declare no conflict of interest.
